# Cross-level attention and lightweight feature extraction for improved tomato ripeness detection in complex greenhouse environments

**DOI:** 10.3389/fpls.2026.1974752

**Published:** 2026-10-30

**Authors:** Xiao Cui, Jisheng Liu, Yujin Guo, Jiayi Li, Enyu He, Kaijie Yang, Fuzhong Li

**Affiliations:** 1 Faculty of Software Technologies, Shanxi Agricultural University, Jinzhong, Shanxi, China; 2 College of Animal Science, Shanxi Agricultural University, Jinzhong, Shanxi, China

**Keywords:** cross-level feature fusion, machine vision, object detection, tomato, YOLO26n

## Abstract

Misclassification of tomato ripeness in greenhouses can lead to premature harvesting, spoilage due to overripeness, and incorrect or missed picks during automated harvesting. However, the color similarity between unripe fruits and leaves, low-contrast changes between adjacent ripeness stages, occlusion by branches and leaves, and the attenuation of small-scale target features make it difficult for existing detection models to simultaneously maintain both ripeness classification accuracy and cross-scenario stability. To address these issues, this paper proposes YOLO26-CUH, an enhanced tomato ripeness detection model based on YOLO26n. This model employs the C2PSA-CC module to jointly model spatial positions and ripeness-related channel responses, thereby suppressing interference from complex backgrounds; it utilizes the C3k2-UIB module to enhance the extraction efficiency of fruit edges, local coloration, and surface textures; further designs the HV-LCA cross-layer feature interaction module to enable selective fusion of shallow-layer spatial details with deep-layer semantic information. This paper constructed a dataset comprising 5, 296 Provence tomato images, classifying the fruits into three maturity levels: unripe, color-changing, and ripe, and employed a separate cherry tomato dataset—which was not used in model training or parameter tuning—for independent external validation. On the Provence tomato test set, YOLO26-CUH achieved precision, recall, mAP@0.50, and mAP@0.50-0.95 of 0.917, 0.896, 0.946, and 0.897, respectively. Compared to the original YOLO26n, these four metrics improved by 6.7, 5.1, 1.9, and 1.9 percentage points, respectively, while the number of model parameters increased only from 2, 375, 421 to 2, 380, 194, and the inference speed reached 93.9 FPS. On the cherry tomato dataset, YOLO26-CUH’s mAP@0.50 and mAP@0.50-0.95 reached 0.808 and 0.656, respectively, representing improvements of 5.3 and 4.1 percentage points over the baseline, with a recall increase of 8.4 percentage points. Luminance perturbation and ablation experiments further demonstrate that this model can enhance the expression of maturity-related features, preserve local details, and reduce missed detections of low-contrast and occluded targets. With the parameter scale remaining largely unchanged, it improves detection accuracy, robustness to luminance perturbations, and generalization performance under an independent cross-domain setting, providing real-time visual support for greenhouse tomato grading and selective harvesting.

## 1 Introduction

Misjudging tomato ripeness directly translates into production losses: harvesting too early results in reduced flavor, color, and market grade, while harvesting too late increases the likelihood of fruit softening, cracking, rotting, and shipping losses. In automated harvesting systems, errors in ripeness identification can also lead to incorrect picking, missed picks, and redundant operations, thereby reducing the efficiency of continuous robotic operations. Therefore, tomato ripeness detection is not merely a visual classification problem but a critical factor affecting harvest yields, storage and transportation losses, and the reliability of automated systems. Greenhouse environments further exacerbate this issue: unripe fruits are similar in color to leaves, and skin color differences are subtle during the color-change stage. Additionally, dense clusters of fruits, foliage obstruction, strong reflections, and shadows can cause the edges and local color information of small targets to be obscured by complex backgrounds. Under these conditions, even if a detection model can identify prominent fruits, it may not be able to reliably distinguish between adjacent ripeness stages (Wang et al., 2022).

In recent years, the YOLO series of models has been widely used for tomato ripeness detection due to their high inference efficiency. Yang et al. (2025) introduced color, channel, and spatial joint attention mechanisms, a bidirectional feature pyramid, and an improved localization loss into YOLOv8n to enhance the representation of ripeness features in complex backgrounds. Sun et al. (2025) developed GCSS-YOLO, which performs fine-grained segmentation of tomatoes at different ripeness stages and supports mobile deployment. Fan and Chai (2026) constructed TRD-Net using SCRConv, SimAM, and MPDIoU, enhancing the feature representation of occluded fruits while reducing redundant computations. Yang and Ju (2025) compared YOLOv5 with an improved YOLOv8, verifying the impact of different detection frameworks on the performance of cherry tomato ripeness recognition. Wang et al. (2024) incorporated Swin Transformer, distribution-shifted convolutions, and Focal-EIoU into YOLOv8 to enhance global context modeling and the ability to localize difficult targets.

Although the above methods improved the accuracy of tomato ripeness detection, their technical approaches mostly focused on adding general-purpose attention mechanisms, replacing convolutional structures, improving feature pyramids, or adjusting localization losses. When ripeness differences primarily manifest as low-contrast color changes, blurred contours, and subtle textural variations, expanding network capacity or further deepening the model does not guarantee that these weak discriminative features will be preserved. Continuous downsampling may further compress skin color transitions, small object edges, and occlusion boundaries, while strong responses generated by complex backgrounds may instead dominate deep-layer features. Therefore, the crux of the current problem is no longer merely extracting more features, but rather how to preserve the subtle features truly related to ripeness amidst background interference.

Subsequent research has begun to focus on improvements in two areas: robustness to complex environments and model deployment efficiency. Zhao et al. (2025) proposed YOLO-DGS, which enhances the ability to distinguish between regular tomatoes and cherry tomatoes by employing segmented convolutions, a streamlined detection layer, and a channel attention mechanism. Wu et al. (2025) developed YOLO-PGC, which utilizes dynamic weight modulation, horizontal–vertical global context, and convolutional inductive feature fusion to mitigate occlusion and background interference. Chen et al. (2025) proposed AFBF-YOLO, which combines ACmix with frequency-aware features for fruit cluster detection and four-level ripeness classification. To address day-night and complex lighting variations, Lu et al. (2026) designed CICE-YOLO, which improves detection stability through color offset correction, heterogeneous convolutions, and multiscale fusion. Zhao et al. (2026) combined a CNN and a Swin Transformer dual backbone in DTBi-YOLOv8-seg to simultaneously achieve ripeness detection and fruit instance segmentation.

These methods have expanded the models’ ability to handle lighting variations, object occlusion, fruits at multiple scales, and pixel-level boundaries; however, two significant bottlenecks remain. On the one hand, complex attention mechanisms, dual backbones, and color preprocessing increase training costs, the number of processing steps, and the difficulty of deployment on edge devices; on the other hand, most feature fusion architectures still rely on concatenation, element-wise addition, or preset weights, lacking selective bidirectional interaction between high-level semantics and low-level color, edges, and textures. Background features may be passed to the detection head alongside target features, while weak edges of small targets and color-changing fruits may be further diluted during the fusion process. Therefore, simply stacking general-purpose modules cannot fundamentally resolve the issue of weak ripeness features being overwhelmed by the background.

In addition to simple ripeness recognition, some studies have combined visual detection with 3D localization and harvesting decisions. Li et al. (2025) proposed a lightweight RAFS-YOLO model and, in conjunction with an RGB-D camera, achieved strawberry ripeness recognition and 3D localization, demonstrating that lightweight detection models can further support spatial decision-making for harvesting robots. Song et al. (2024) combined FastMLCA-YOLOv8 with stem segmentation, skeleton extraction, and depth compensation to achieve the localization of harvesting points on tomato clusters. Wang et al. (2025) proposed YOLO-TMPPD, which simultaneously detects tomato ripeness and key harvesting points. Chen et al. (2024) used MTD-YOLO to jointly output the position and ripeness of individual fruits, as well as the position and ripeness of the fruit cluster.

The aforementioned studies have advanced the extension of visual detection from ripeness recognition to robotic operation decision-making; however, 3D localization, key point extraction, and fruit stalk analysis typically rely on specific depth-sensing devices or multi-stage post-processing, which cannot replace the effective modeling of weak color and edge features performed during the ripeness detection stage. At the same time, most studies still conduct training and testing on data generated from the same variety, the same greenhouse, or the same harvesting equipment. Since the internal test set and training set share similar data distributions, high internal detection accuracy does not necessarily indicate that the model can adapt to different fruit shapes, sizes, skin textures, and growing environments.

To address the central challenge that weak ripeness-related color, texture, and edge features may be disturbed, attenuated, or lost during feature extraction and multiscale fusion in complex greenhouse backgrounds, this paper develops YOLO26-CUH based on YOLO26n. The proposed architecture introduces three complementary components at different stages of the feature-processing pipeline. C2PSA-CC enhances ripeness-related feature responses through spatial attention and channel modulation; C3k2-UIB improves local feature extraction while preserving fine-grained edge and texture information; and HV-LCA promotes selective interaction between shallow spatial details and deep semantic features during multiscale fusion. These components are therefore designed around the same objective of preserving and transmitting weak ripeness-discriminative information throughout the network.

This paper constructs a dataset comprising 5, 296 images of Provence tomatoes across three maturity stages, covering various lighting conditions, occlusion levels, object scales, and growth postures, and classifies the fruits into three stages: unripe, color-changing, and ripe. In addition to internal training, validation, and testing, independent external validation was conducted using cherry tomato images that were not used in training, threshold selection, or parameter tuning to assess the model’s generalization ability under varying fruit shapes, sizes, and varieties. Through baseline comparisons, module-by-module ablation, testing under representative complex scenarios, independent cross-domain validation, and Grad-CAM++ feature-response visualization, we verified the role of C2PSA-CC in background suppression and channel response enhancement, the ability of C3k2-UIB to preserve weak edges and local details, and the contribution of HV-LCA in linking shallow-level details with deep-level semantics. Thus, the proposed architecture is organized around a common objective: preserving and effectively transmitting weak ripeness-related color, texture, and edge information under complex greenhouse conditions.

## 2 Materials and methods

### 2.1 Self-constructed tomato ripeness grading dataset

This study was conducted from May 9 to June 6, 2026, in Yuci District, Jinzhong City, Shanxi Province, to collect tomato images. The geographic location of the study area and the greenhouse setting are shown in **Figure 1**. The experimental variety was the Provence tomato. The experimental plots were cultivated in plastic-covered greenhouses with a row spacing of 1.5 m; each row contained two rows of plants, with a plant spacing of 35 cm. Plants that were healthy and free of obvious pests, diseases, or mechanical damage were selected for imaging. Standardized imaging equipment and a consistent data collection protocol were used to minimize the impact of variations in equipment settings and imaging methods on data quality.

**Figure 1:**
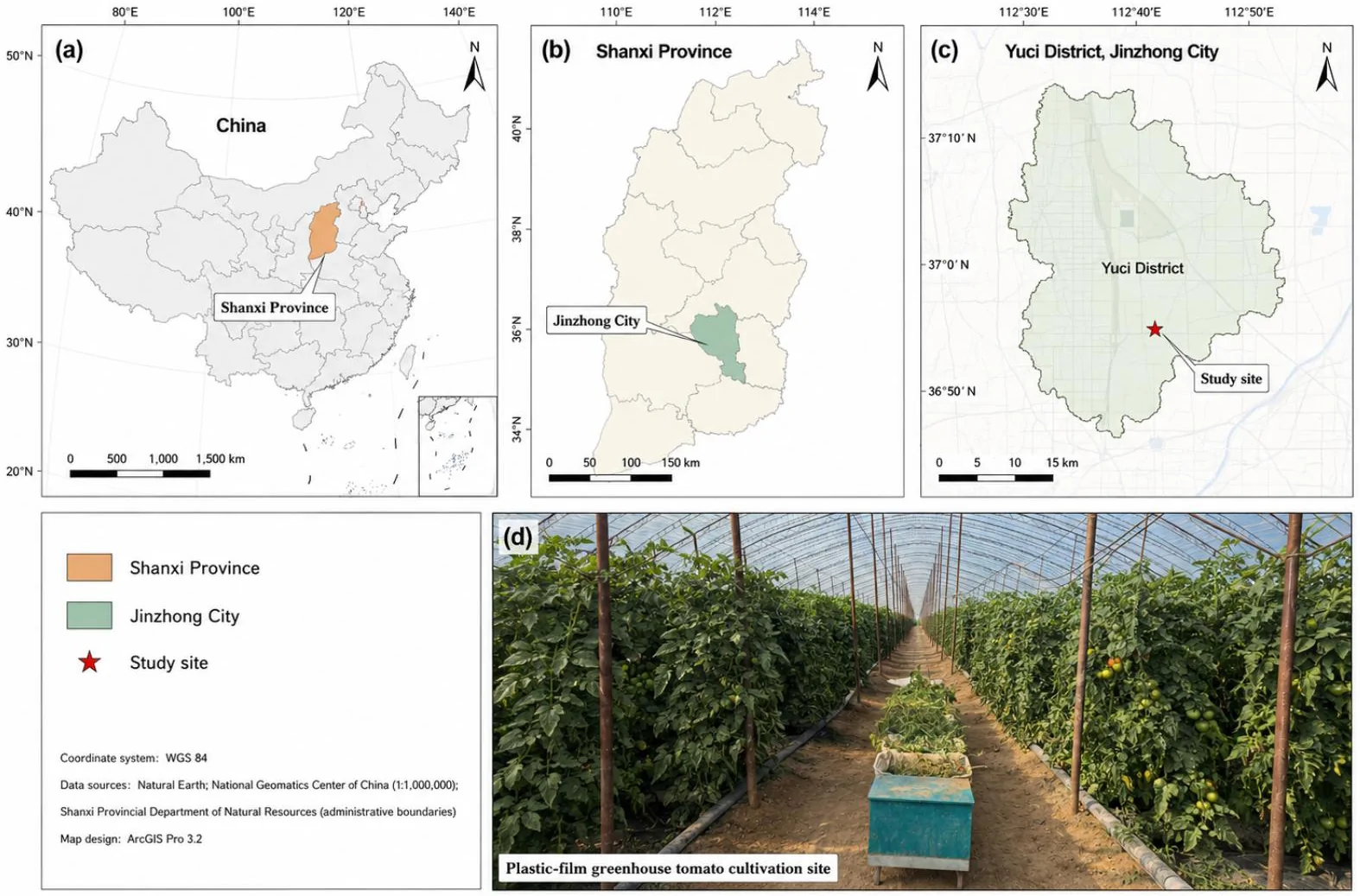
Geographic location of the study area and overview of the greenhouse tomato farm. **(a)** Location of Shanxi Province in China; **(b)** location of Jinzhong City in Shanxi Province; **(c)** location of Yuci District in Jinzhong City; and **(d)** interior view of the plastic-film greenhouse tomato farm.

During the experiment, a total of four image acquisitions were conducted, with an interval of approximately one week between each acquisition. By adjusting the shooting distance, angle, and lighting direction, tomato images were captured under various natural lighting conditions. This ensured that the dataset covered typical greenhouse scenarios—such as foliage obstruction, fruit overlap, dense targets, uneven lighting, and complex backgrounds—thereby realistically reflecting the environmental disturbances encountered during intelligent field inspections and providing a data foundation for training and evaluating tomato ripeness detection models.

A total of 5, 500 raw tomato images were captured for this study. After excluding invalid images—such as those that were severely blurred, overexposed, had significantly missing parts of the target, or where ripeness could not be accurately determined—5, 296 valid images were ultimately selected. Unripe fruits were predominantly green; color-changing fruits exhibited a mixture of green with yellow, orange, or light-red regions; and ripe fruits were predominantly red. Because tomato ripening is a continuous process and adjacent stages are not always visually distinct, these criteria were used as practical annotation rules rather than strict pixel-level color thresholds. Annotations were initially performed by one trained annotator and subsequently reviewed by a second researcher. Samples with disagreements in class assignment or bounding-box placement were jointly discussed until a consensus was reached. Samples whose ripeness stage remained uncertain after joint review were treated as ambiguous and were not assigned a maturity label. Using the LabelImg tool, tomato fruits that were fully visible or only slightly obscured by branches and leaves were annotated individually as “unripe, “ “color-changing, “ or “ripe, “ and the annotation files were saved in YOLO format. Because a single greenhouse image commonly contains multiple fruits with varying degrees of occlusion, including both fully visible and heavily occluded ones, we excluded ineligible targets at the object level rather than discarding the entire image. Fruits that were severely occluded, morphologically incomplete, or for which the ripeness level could not be reliably determined were not annotated. The distribution of images and labels across each ripeness level is shown in **Figure 2**.

**Figure 2:**
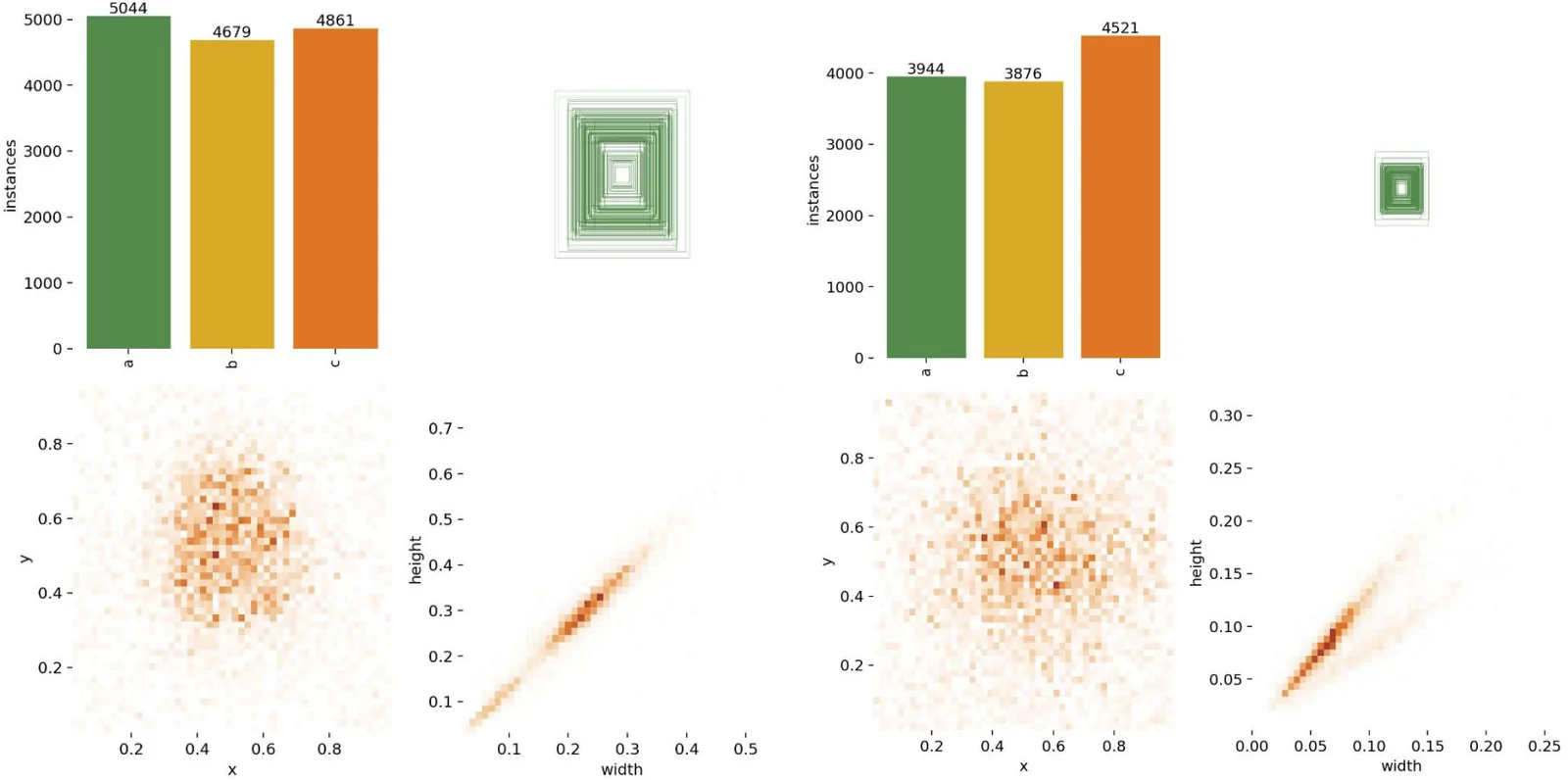
Sample statistics and distribution of annotation features for the greenhouse tomato ripeness detection dataset.

Each image was coded with its acquisition session, plant ID, camera angle, and shooting distance. To avoid information leakage caused by highly correlated images, the dataset was not split by random image-level sampling. Instead, partitioning was performed at the plant-group level. The dataset was first stratified by acquisition session, after which all images of the same plant ID, regardless of camera angle, shooting distance, or acquisition session, were assigned as a whole to a single subset. No plant ID was shared across the training, validation, and test sets. The resulting training, validation, and test sets contained 4, 232, 532, and 532 images, respectively, in an approximate ratio of 8:1:1, with all four acquisition sessions represented in each subset.

To further evaluate the generalization ability of the model under domain shifts involving tomato variety, fruit size, morphology, and environmental conditions, this study adopted the Busan 88 cherry tomato dataset as an independent external validation set. This dataset was independently collected by the authors for the present study and was not obtained from a public dataset or repository. A total of 804 images were captured from greenhouses in Yuci District, Jinzhong City, Shanxi Province, China, using a Xiaomi 14 smartphone at a resolution of 4000 × 3000 pixels. After removing 67 blurred images that could not be reliably annotated, 737 images were retained for validation. The samples were collected from different greenhouses at the same experimental site with a single-row cultivation pattern, where the row spacing was 1 m and the plant spacing was 30 cm. Substantial differences exist between this dataset and the self-built Provence tomato dataset in fruit size, fruit shape, peel coloration, cultivation background, and image distribution. Each fruit instance was annotated into one of three ripeness categories (unripe, color-changing, and ripe) following the same labeling protocol as the self-built dataset. In total, 12, 341 annotated fruit instances were obtained, including 3, 944 unripe, 3, 876 color-changing, and 4, 521 ripe instances. This external dataset was not used for model training, hyperparameter optimization, or confidence-threshold tuning. The model trained on the self-built dataset was directly applied for inference to evaluate cross-domain generalization under changes in tomato variety, fruit morphology, target scale, and environmental conditions.

### 2.2 Methods

Plastic-film greenhouses present complex detection challenges for tomato ripeness identification. These challenges include fruit occlusion by branches and leaves, target overlap, variable light interference, subtle color differences between adjacent ripening stages, easy loss of small-object features, and insufficient cross-layer feature fusion (Chen et al., 2024). This paper therefore proposes an improved tomato ripeness detection network, YOLO26-CUH. #The overall architecture of YOLO26-CUH is shown in **Figure 3**. First, a C2PSA-CC feature enhancement module is constructed. This module utilizes CAA spatial attention to model long-range dependencies in both horizontal and vertical directions, and employs CGMA channel attention to strengthen feature responses related to fruit skin color, texture, and ripeness, thereby improving the model’s ability to focus on fine-grained ripeness features. Second, we adopt the universal inverted bottleneck (UIB) structure to reconstruct the C3k2 module, forming the C3k2-UIB lightweight feature extraction module. This reduces redundant computations while enhancing the model’s ability to represent tomato edges, localized colored regions, and occluded small objects. Finally, we introduce the HV-LCA cross-layer feature interaction enhancement module into the neck network. Through channel alignment, cross-attention, and intensity enhancement mechanisms, this module adaptively fuses shallow-layer spatial details with deep-layer semantic information, thereby improving the transmission and fusion of multiscale features. The three modules are arranged sequentially in the feature-processing pipeline. C2PSA-CC first enhances ripeness-related feature responses in the backbone; C3k2-UIB then extracts local features while preserving fine-grained details; and HV-LCA finally integrates shallow spatial information with deep semantic features during neck-stage multiscale fusion. They correspond respectively to feature enhancement, local extraction, and cross-level interaction.These improvements collectively enhance YOLO26n’s detection performance and generalization capability for tomatoes at different stages of ripeness in complex greenhouse environments, while maintaining a parameter scale comparable to that of the baseline.

**Figure 3:**
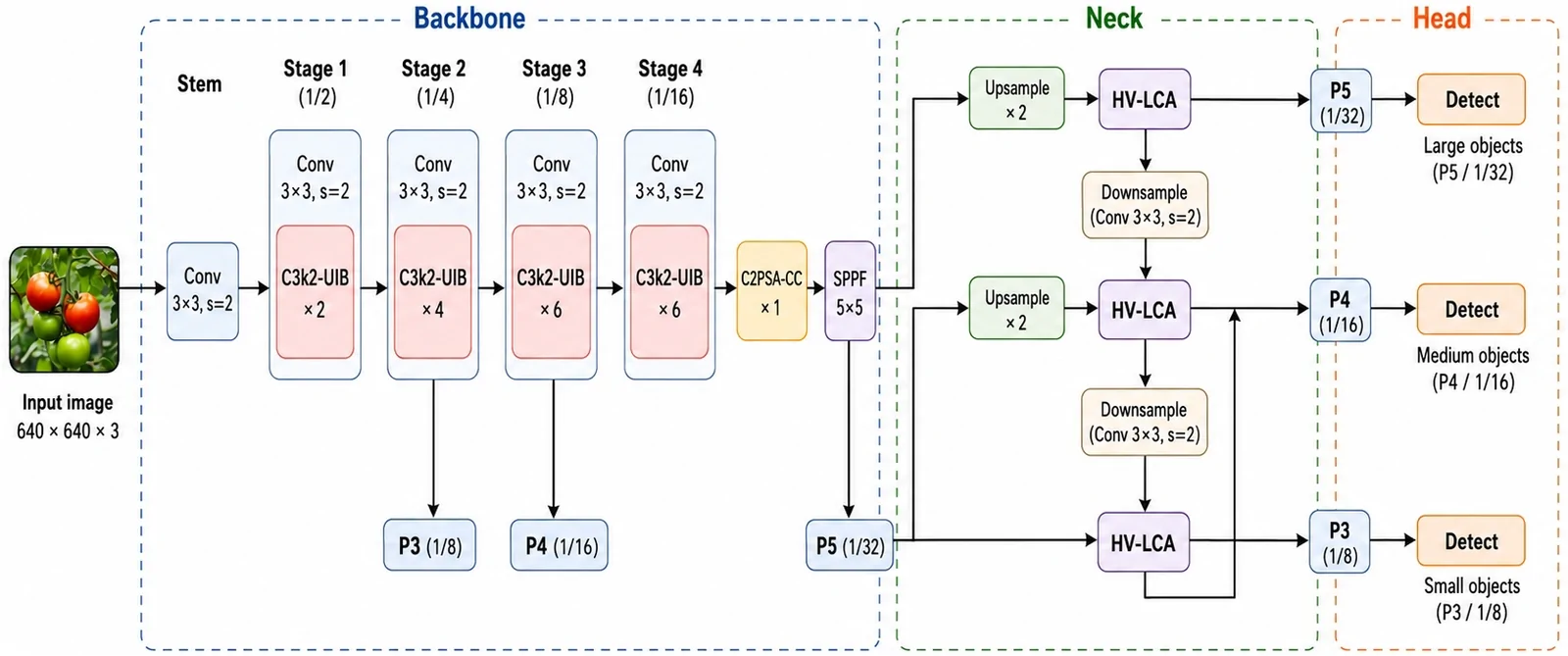
Overall architecture of the improved YOLO26-CUH tomato ripeness detection network.

#### 2.2.1 C2PSA-CC

As tomatoes ripen, chlorophyll gradually breaks down while pigments such as carotenoids continue to accumulate, causing the fruit skin color to change from green to yellow, orange, and red. Therefore, the primary visual differences among different ripeness levels are concentrated in the fruit skin color and the proportion of localized coloring; however, changes in light conditions inside the greenhouse, shading from branches and leaves, and complex backgrounds can diminish these differences. The C2PSA module in the original YOLO26n lacks the ability to selectively enhance color cues specific to ripeness, making it prone to mistaking color changes caused by lighting for differences in ripeness, which leads to confusion between adjacent ripeness levels. To address this, this paper combines CAA spatial attention with a self-designed CGMA channel modulation mechanism to construct the C2PSA-CC module, enabling collaborative modeling of fruit region localization and ripeness feature enhancement.

CAA first aggregates local context using 7×7 average pooling and establishes long-range dependencies in the horizontal and vertical directions through 1 × 11 and 11 × 1 depthwise convolutions. Since tomato color features are only reliable for ripeness classification after the fruit region has been accurately localized, CAA first highlights the fruit outline, shoulder, and localized colored areas while suppressing responses from branches, leaves, and the greenhouse background, thereby providing relatively clean target features for subsequent color-related channel modeling.

Building on this, this paper designs CGMA to address the relationship where changes in ripeness cause changes in fruit peel color, which in turn manifest as differences in the responses of feature channels. This module first uses global average pooling to calculate the response intensity of each channel across the entire fruit region, then employs two layers of 1×1 convolutions to learn the nonlinear dependencies between channels and generate adaptive channel weights. Since certain feature channels are more sensitive to coloring states such as green, yellow, orange, and red, CGMA enhances channels related to fruit peel color, coloring proportion, and ripeness texture, while simultaneously attenuating irrelevant responses caused by leaves, soil, trellises, and local highlights. Therefore, rather than directly judging ripeness based on fixed color thresholds, CGMA autonomously learns the channel response patterns corresponding to different ripeness levels from training data, thereby improving the stability of ripeness classification under complex lighting conditions.

In C2PSA-CC, the input features are split into two branches after a 1×1 convolution: one branch retains the original shallow-level information, while the other is fed into the PSABlock-CC for enhancement. The enhancement branch first uses CAA to identify areas of interest, then relies on CGMA to determine which ripeness-related channels require amplification, forming a processing workflow that seamlessly integrates spatial localization with color representation. The module further introduces learnable fusion coefficients to adaptively adjust the contribution of the CGMA branch, thereby preventing color responses from being overly influenced by lighting noise during the early stages of training. Finally, the two feature streams are fused through concatenation and a 1×1 convolution, thereby enhancing YOLO26n’s ability to classify and localize occluded objects, small targets, and tomatoes of adjacent maturity levels. The structure of the C2PSA-CC module is illustrated in **Figure 4**.

**Figure 4:**
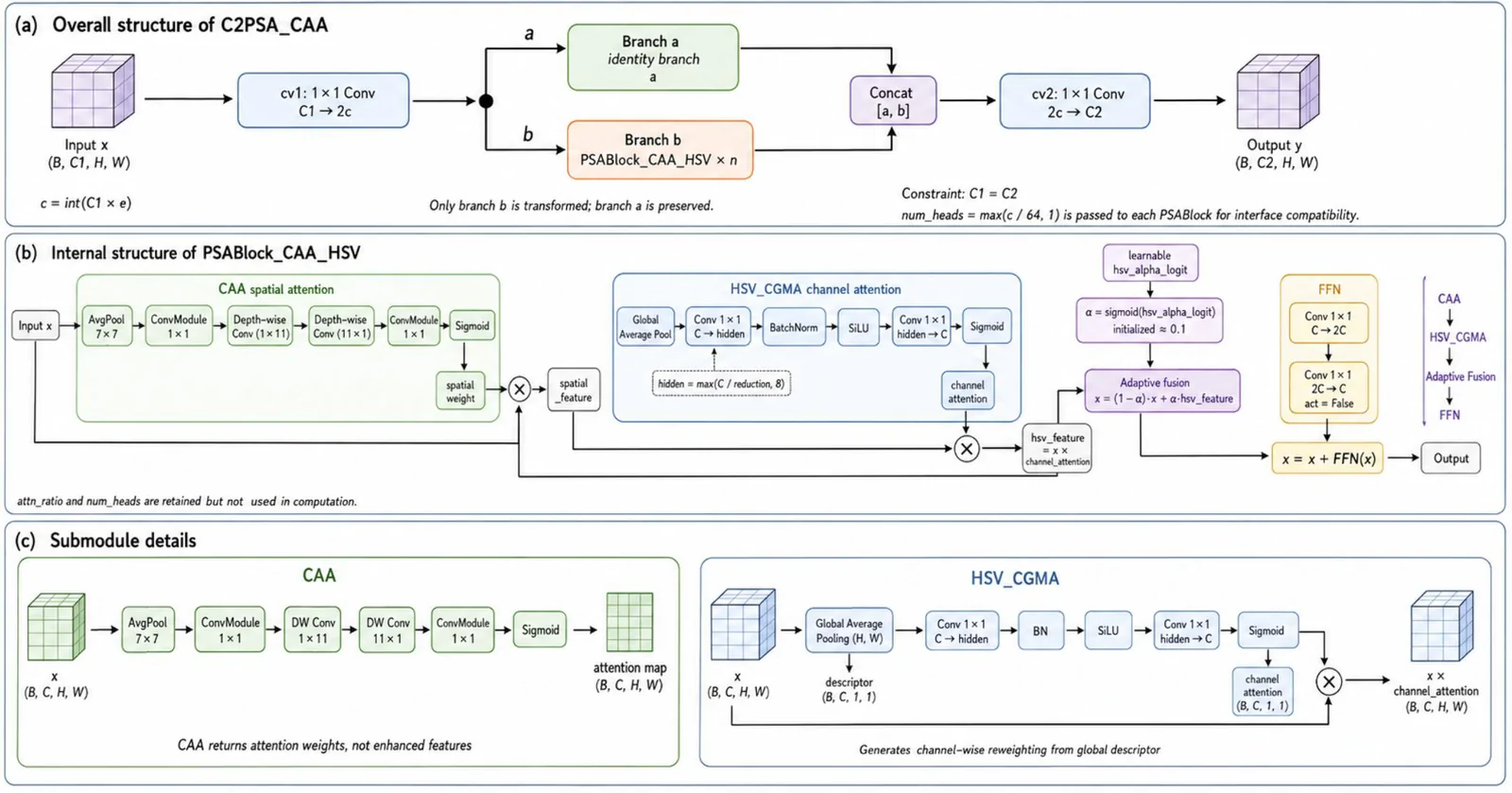
Structure of the C2PSA-CC feature enhancement module.

#### 2.2.2 C3k2-UIB lightweight feature extraction module

Building on the enhanced ability of the aforementioned C2PSA-CAA-CGMA module to focus on key regions and ripeness features of tomatoes, the model still needs to further improve its efficiency in extracting local details of fruit edges, surface textures, and occluded areas. The C3k2 module in the original YOLO26n primarily uses a conventional bottleneck for feature transformation; however, standard convolutions tend to generate excessive redundant computations in complex greenhouse scenes, making it difficult to balance detection accuracy with lightweight deployment. To address this, this paper introduces the Universal Inverted Bottleneck (UIB) structure to replace the conventional feature extraction units within the C3k2 module, thereby constructing the C3k2-UIB lightweight feature enhancement module to improve the network’s ability to characterize dense, occluded, and small-scale tomato targets.

The UIB first uses 3×3 deep convolutions to independently extract local spatial information from each channel, followed by 1×1 convolutions to perform channel mapping, adjusting the number of intermediate channels to an integer multiple of 8 to improve computational efficiency. Subsequently, the module employs a second 3×3 deep convolution to further capture fine-grained features such as tomato contours, local color regions, and surface textures, and finally restores the output channels via 1×1 linear projection. This architecture decouples spatial feature extraction from channel information interaction; while expanding the modeling capability for local features, it helps reduce the parameter and computational redundancy generated by standard convolutions.

In the improved C3k2 architecture, the input features first undergo channel adjustment via a 1×1 convolution and are then split into two branches along the channel dimension. One branch retains the original shallow-level information, while the other sequentially passes through multiple UIB units for deep feature extraction. The features generated at each stage are concatenated with the retained branch, and channel fusion is finally achieved through a 1×1 convolution. This architecture fully reuses feature information from different depths, minimizes the loss of tomato small-object edge and texture features during the continuous propagation process, and complements the aforementioned C2PSA-CAA-CGMA module, thereby further enhancing the accuracy of tomato ripeness detection and the efficiency of feature extraction for YOLO26n in complex greenhouse environments. The structure of the C3k2-UIB module is shown in **Figure 5**.

**Figure 5:**
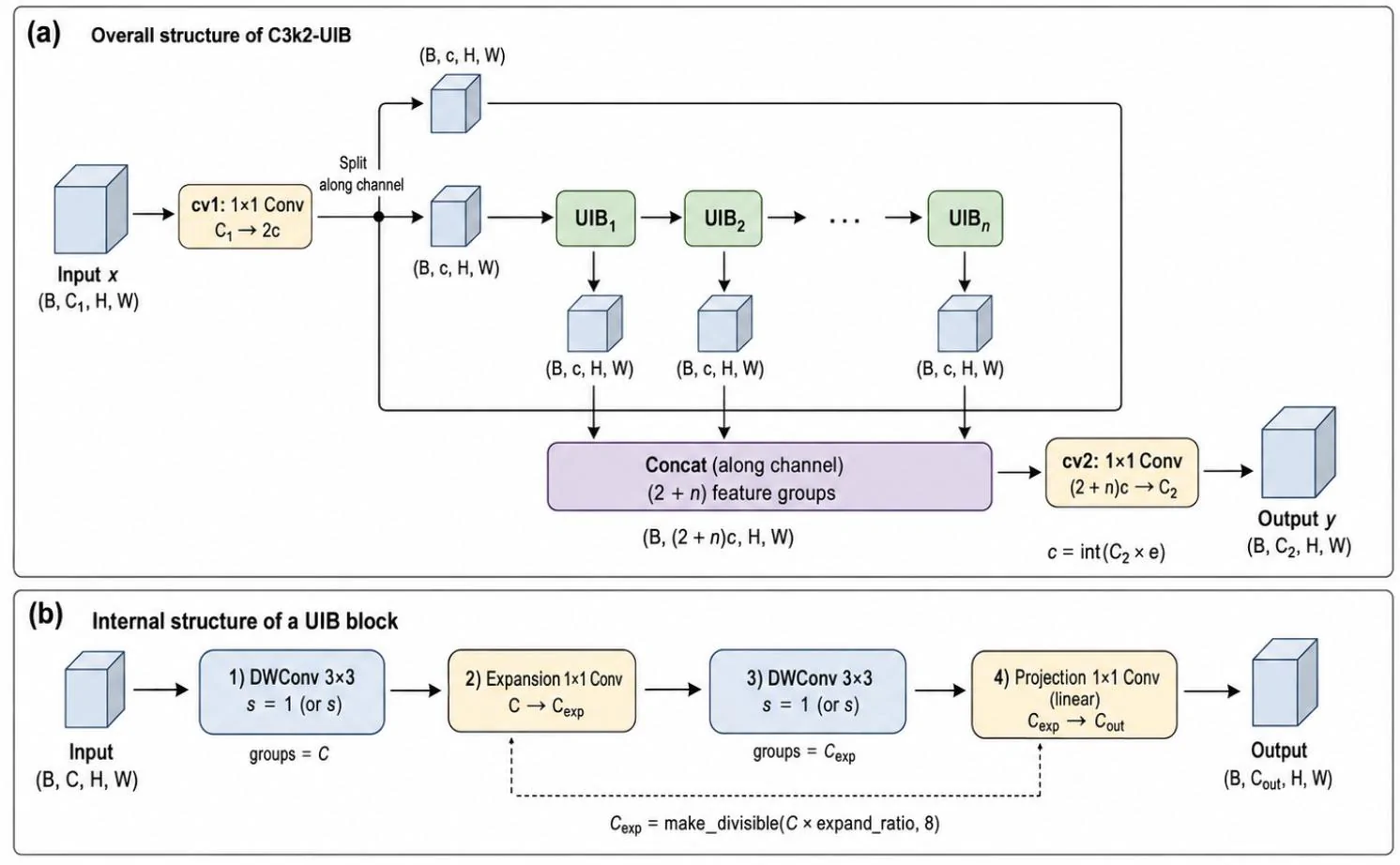
Structure of the C3k2-UIB lightweight feature extraction module. **(a)** Overall structure of the C3k2-UIB module; **(b)** internal structure of the UIB block.

#### 2.2.3 HV-LCA inter-layer feature interaction enhancement module

Although the C2PSA-CAA-CGMA approach enhances the focus on maturity-related features and the C3k2-UIB approach improves the efficiency of local detail extraction, there remains an issue of insufficient integration between semantic information and spatial details across different network layers. Shallow-layer features contain rich information about tomato edges, textures, and local coloration, while deep-layer features possess stronger semantic representation capabilities for the target; simply concatenating or summing these features makes it difficult to fully establish a correlation between them. To address this, this paper designs the HV-LCA cross-layer feature interaction enhancement module, which achieves adaptive fusion of features from different layers through channel alignment, cross-attention, and detail enhancement, thereby improving the model’s ability to recognize tomato targets that are occluded, densely clustered, or at similar stages of ripeness.

HV-LCA first uses 1×1 convolutions to map the two input feature streams to the same channel dimension and standardizes the feature distributions via LayerNorm. Subsequently, the cross-attention module generates a query matrix from the first feature stream and a key-value matrix from the second feature stream; the query, key, and value matrices all undergo 1×1 convolutions and 3×3 deep convolutions, introducing local spatial information while reducing computational overhead. The features are divided into multiple attention heads and normalized. A learnable temperature coefficient is used to calculate the cross-channel correlation between the two feature streams, enabling the high-level semantic information in the second feature stream to adaptively supplement the local details in the first feature stream. The cross-attention results are projected via a 1×1 convolution and fused with the original features in a residual manner to preserve the original information and improve training stability.

After completing cross-layer feature interaction, the module further introduces an Intensity Enhancement Layer (IEL) to refine the fused features. The IEL first expands the channels using a 1×1 convolution and divides the features into two streams via a 3×3 deep convolution; these two feature streams are enhanced through deep convolution and Tanh nonlinearity, respectively, before undergoing element-wise multiplication to achieve selective gating of effective responses. Finally, the channel dimension is restored via a 1×1 convolution, and the result is fused with the cross-attention output through a residual connection. This module simultaneously leverages high-level semantic information and low-level fine-grained features to enhance the representation of tomato contours, local color variations, and occlusion boundaries, thereby further improving the performance of YOLO26n in detecting tomato ripeness within complex greenhouse environments. The structure of the HV-LCA module is shown in **Figure 6**.

**Figure 6:**
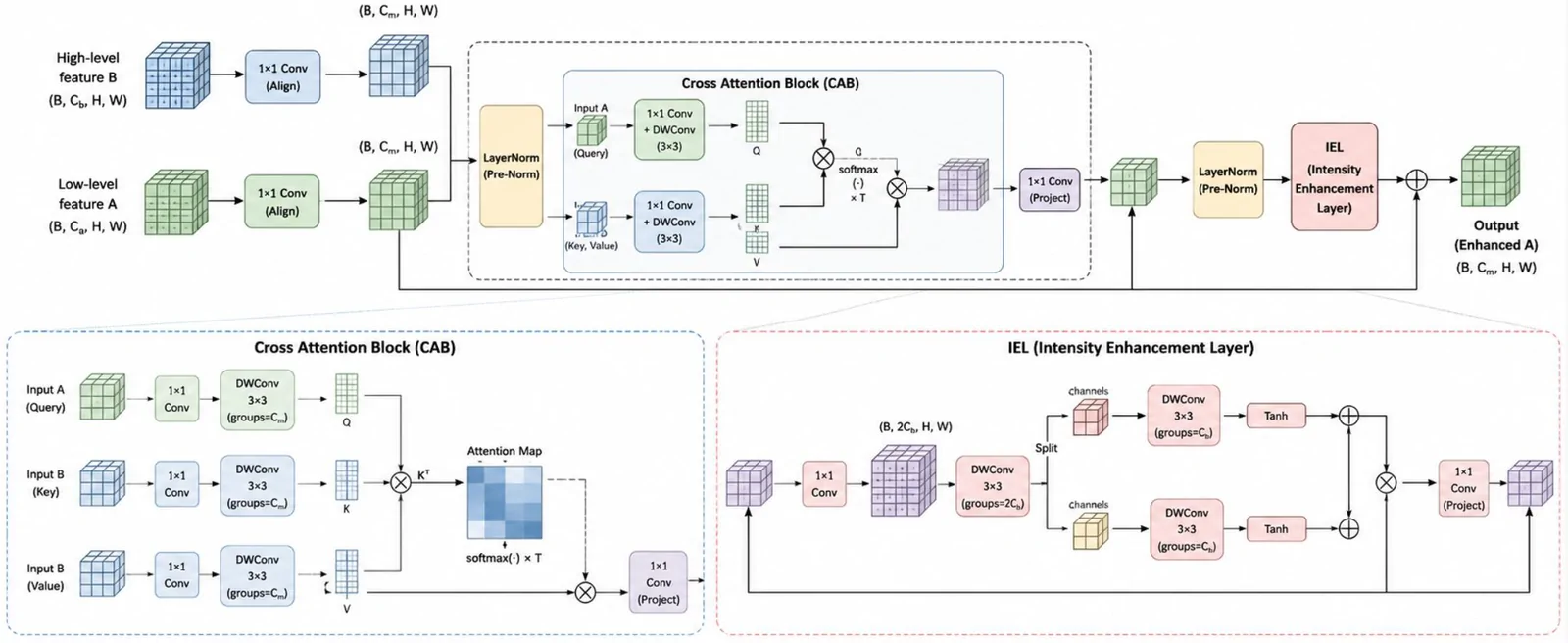
Structure of the HV-LCA cross-layer feature interaction enhancement module.

### 2.3 Evaluation metrics

To comprehensively evaluate model performance, this paper selects precision (P), recall (R), mAP@0.50, mAP@0.50-0.95, the number of parameters, GFLOPs, and FPS as evaluation metrics. P and R reflect false positives and false negatives, respectively, while mAP@0.50 and mAP@0.50-0.95 are used to measure detection accuracy at different intersection-to-union ratio thresholds. The number of parameters and GFLOPs are used to evaluate the model’s scale and computational complexity, while FPS is used to measure real-time inference capability. For FPS testing, the batch size was set to 1, the input size to 640×640, and half-precision inference on a GPU was used; after 500 warm-up runs, 2, 000 latency tests were conducted, repeated independently five times, and the results were averaged. The above metrics evaluate the comprehensive performance of different models in terms of detection accuracy, model lightweightness, and real-time performance.

### 2.4 Experimental environment

To ensure fairness in comparisons across different models and ablation configurations, all models in this paper were trained and tested under the same hardware and software environment, data split, and training strategy. The experimental platform consisted of a high-performance desktop workstation equipped with an Intel Core i9 processor, 32 GB of RAM, a 1 TB solid-state drive, and an NVIDIA GeForce RTX 4090 GPU. The models were implemented using the PyTorch 2.2.0 deep learning framework, with CUDA 11.8 as the GPU computing environment. All input images were uniformly resized to 640 × 640 pixels using proportional scaling and edge padding. The model was trained for 300 epochs with a batch size of 6 and 4 data-loading threads. During training, the optimal model weights were saved based on detection performance on the validation set, and these weights were used to evaluate the test set.

Model performance was evaluated using precision, recall, mAP@0.50, and mAP@0.50-0.95, while model scale and computational complexity were measured by the number of parameters and GFLOPs. Inference speed was tested uniformly on an RTX 4090 GPU, with input image dimensions set to 640 × 640 pixels, a batch size of 1, and half-precision floating-point inference. Prior to official timing, the model was warmed up for 500 iterations, followed by 2, 000 consecutive inference tests; this process was repeated independently five times, and the frames per second were calculated based on the average results to minimize the impact of GPU initialization, caching, and random latency fluctuations on the test results.

SGD was adopted as the optimizer with a momentum of 0.937 and a weight decay of 0.0005. A linear learning-rate scheduler was used, with an initial learning rate (lr0) of 0.01 and a final learning-rate fraction (lrf) of 0.005. A warm-up strategy of 3 epochs was adopted; the warm-up momentum was set to 0.8 and the bias warm-up learning rate was 0.1. The model was trained from scratch without pretrained weights. During validation and testing, the confidence threshold was set to 0.001 and the NMS IoU threshold was set to 0.7. Data augmentation included HSV augmentation, translation, scaling, horizontal flipping, and Mosaic augmentation. For the main experiments, the random seed was fixed to 1 and deterministic mode was enabled to ensure reproducibility. To assess run-to-run variability, all eight ablation configurations were additionally trained using three independent random seeds under identical data splits and training settings. Precision, recall, mAP@0.50, and mAP@0.50–0.95 are reported as mean ± standard deviation across the three runs.

## 3 Experimental results and analysis

### 3.1 Overall detection performance

**Table 1** shows the overall performance of YOLO26-CUH on the Provence Tomato test set, as well as the detection results for different ripeness categories. The model achieved precision, recall, mAP@0.50, and mAP@0.50-0.95 values of 0.917, 0.896, 0.946, and 0.897, respectively. Under the same hardware and inference settings, the model has 2, 380, 194 parameters, a computational load of 6.2 GFLOPs, and an inference speed of 93.9 FPS, indicating that the proposed model can meet the basic requirements for real-time tomato ripeness detection while maintaining a relatively small model size.

**Table 1:** Summary of experimental results.

Classes	P	R	mAP@0.50	mAP@0.50-0.95	Parameters	GFLOPs	FPS
all	0.917	0.896	0.946	0.897	2, 380, 194	6.2	93.9
Unripe	0.949	0.886	0.925	0.889			
Color-changing	0.855	0.911	0.957	0.903			
Ripe	0.947	0.891	0.957	0.901			

Looking at the results by ripeness category, unripe tomatoes had the highest precision, reaching 0.949, but their mAP@0.50 was 0.925, which was lower than those of the color-changing and ripe categories. These results indicate that the model’s predictions for tomatoes identified as unripe are generally reliable, but some unripe fruits are still prone to being missed. This may be related to the similarity in color and local texture between unripe fruits and surrounding leaves, which makes the fruit edges less distinct against the complex green background.

Tomatoes in the color-changing stage achieved the highest recall of 0.911, while their mAP@0.50 and mAP@0.50-0.95 reached 0.957 and 0.903, respectively. However, the precision for this category was 0.855, which was lower than that of the other two ripeness categories, indicating that tomatoes in the color-changing stage were more likely to be confused with those in adjacent ripeness stages. Since the color-changing stage represents an intermediate state in the continuous transition from unripe to ripe, the fruit skin often exhibits areas of green, yellow, or light red simultaneously, resulting in relatively blurred class boundaries.

For ripe tomatoes, the precision, recall, mAP@0.50, and mAP@0.50-0.95 were 0.947, 0.891, 0.957, and 0.901, respectively, indicating relatively balanced performance across the metrics. Overall, the differences in mAP@0.50-0.95 among the three tomato ripeness categories were small, indicating that YOLO26-CUH maintained relatively stable bounding-box localization performance across different ripeness categories.

### 3.2 Comparison with contemporary detection models

To further evaluate the performance of YOLO26-CUH, quantitative comparisons were conducted with YOLOv8n, YOLOv9t, YOLOv10n, YOLO11n, YOLO12n, RT-DETR, and YOLO26n under the same experimental conditions. Precision, recall, mAP@0.50, mAP@0.50–0.95, parameter number, GFLOPs, and FPS were compared, as summarized in **Table 2**.

**Table 2:** Quantitative comparison of different detection models.

Model	P	R	mAP@0.50	mAP@0.50–0.95	Parameters	GFLOPs	FPS
YOLOv8n	0.927	0.829	0.932	0.874	3, 006, 233	8.1	185.1
YOLOv9t	0.882	0.834	0.935	0.886	1, 971, 369	7.6	68.3
YOLOv10n	0.857	0.867	0.926	0.870	2, 695, 586	8.2	141.7
YOLO11n	0.917	0.846	0.935	0.884	2, 582, 737	6.3	164.6
YOLO12n	0.908	0.858	0.935	0.886	2, 508, 929	5.8	117.2
RT-DETR	0.856	0.754	0.844	0.762	16, 742, 209	42.6	69.0
YOLO26n	0.850	0.845	0.927	0.878	2, 375, 421	5.2	114.2
YOLO26-CUH	0.917	0.896	0.946	0.897	2, 380, 194	6.2	93.9

As shown in **Table 2**, YOLO26-CUH achieved the highest mAP@0.50 and mAP@0.50–0.95 among the compared models while maintaining a parameter count comparable to the YOLO26n baseline, although its computational cost and inference speed were moderately affected.

### 3.3 Ablation experiments for the proposed module

To quantitatively examine the individual and combined effects of C2PSA-CC, C3k2-UIB, and HV-LCA—we conducted a comprehensive ablation study under identical data split, training parameters, and inference conditions. For the sake of clarity, we refer to C2PSA-CC, C3k2-UIB, and HV-LCA as modules A, B, and C, respectively. All eight possible combinations of the three modules were evaluated through repeated independent training runs under identical data splits, training parameters, and inference conditions. Precision, recall, mAP@0.50, and mAP@0.50–0.95 are reported as mean ± standard deviation in **Table 3**.

**Table 3:** Ablation results reported as mean ± standard deviation across three independent runs.

Model	Module			P (mean ± SD)	R (mean ± SD)	mAP@0.50 (mean ± SD)	mAP@0.50–0.95 (mean ± SD)
	A	B	C				
1				0.848 ± 0.00529	0.845 ± 0.00312	0.929 ± 0.00207	0.880 ± 0.00372
2	✓			0.888 ± 0.00549	0.873 ± 0.00360	0.946 ± 0.00490	0.891 ± 0.00309
3		✓		0.864 ± 0.00512	0.875 ± 0.00424	0.931 ± 0.00504	0.879 ± 0.00273
4			✓	0.874 ± 0.00253	0.898 ± 0.00235	0.944 ± 0.00292	0.894 ± 0.00202
5	✓	✓		0.870 ± 0.00240	0.880 ± 0.00597	0.926 ± 0.00310	0.877 ± 0.00469
6	✓		✓	0.911 ± 0.00703	0.887 ± 0.00423	0.937 ± 0.00511	0.883 ± 0.00543
7		✓	✓	0.917 ± 0.00631	0.825 ± 0.00367	0.929 ± 0.00398	0.887 ± 0.00414
8	✓	✓	✓	0.917 ± 0.00319	0.898 ± 0.00399	0.948 ± 0.00136	0.898 ± 0.00288

Across the repeated runs, the YOLO26n baseline achieved precision, recall, mAP@0.50, and mAP@0.50–0.95 values of 0.848 ± 0.00529, 0.845 ± 0.00312, 0.929 ± 0.00207, and 0.880 ± 0.00372, respectively. After C2PSA-CC was introduced, the corresponding values increased to 0.888 ± 0.00549, 0.873 ± 0.00360, 0.946 ± 0.00490, and 0.891 ± 0.00309, respectively. Compared with the baseline, the mean precision, recall, mAP@0.50, and mAP@0.50–0.95 increased by 4.0, 2.8, 1.7, and 1.1 percentage points, respectively. These results indicate that C2PSA-CC provides the largest mean mAP@0.50 improvement among the three individually evaluated modules.

When C3k2-UIB was used independently, the number of model parameters decreased from 2, 375, 421 to 2, 209, 845, representing a reduction of 165, 576 parameters, while the computational load decreased from 5.2 to 4.9 GFLOPs. With C3k2-UIB alone, the model kept detection performance close to the baseline. Precision, recall, mAP@0.50, and mAP@0.50–0.95 were 0.864 ± 0.00512, 0.875 ± 0.00424, 0.931 ± 0.00504, and 0.879 ± 0.00273, respectively. Compared with the baseline, the mean mAP@0.50 rose by 0.2 percentage points, while the mean mAP@0.50–0.95 dropped by 0.1. In other words, C3k2-UIB mainly removes model redundancy rather than boosting detection accuracy on its own.

When HV-LCA was introduced alone, it achieved precision, recall, mAP@0.50, and mAP@0.50–0.95 values of 0.874 ± 0.00253, 0.898 ± 0.00235, 0.944 ± 0.00292, and 0.894 ± 0.00202, respectively. Compared with the baseline, the mean recall increased by 5.3 percentage points, representing the largest recall improvement among the three standalone modules. The mean mAP@0.50 and mAP@0.50–0.95 increased by 1.5 and 1.4 percentage points, respectively. These results suggest that HV-LCA contributes primarily to improved recall and overall detection performance. However, HV-LCA increased the number of model parameters to 2, 558, 041 and the computational load to 6.5 GFLOPs.

No pairwise combination of the three modules consistently outperformed the corresponding single-module configurations. For example, the A + B combination achieved a mean mAP@0.50 of 0.926 ± 0.00310, which was slightly lower than the baseline value of 0.929 ± 0.00207 and also failed to surpass the result obtained using module A alone. This finding suggests that the effects of the three modules are not simply additive and that certain combinations may alter the balance among channel enhancement, feature compression, and cross-layer feature fusion. The observed performance degradation may be associated with changes in feature distributions introduced by different modules. For example, feature reweighting by C2PSA-CC and feature compression by C3k2-UIB may alter the information passed to subsequent layers, potentially reducing the complementarity of some pairwise configurations.

After A, B, and C were simultaneously incorporated, YOLO26-CUH achieved the best overall mean performance across the repeated runs, with precision, recall, mAP@0.50, and mAP@0.50–0.95 values of 0.917 ± 0.00319, 0.898 ± 0.00399, 0.948 ± 0.00136, and 0.898 ± 0.00288, respectively. Compared with the YOLO26n baseline, the corresponding mean values increased by 6.9, 5.3, 1.9, and 1.8 percentage points, respectively. The number of model parameters increased by only 4, 773, corresponding to approximately 0.20%, while the computational complexity increased from 5.2 to 6.2 GFLOPs and the inference speed decreased from 114.2 to 93.9 FPS.

### 3.4 Comparative experiments: validation of the hierarchical architecture of C3k2-UIB

To determine the optimal placement of C3k2-UIB across different feature levels, hierarchical replacement experiments were conducted on layers P2, P3, P4, and P5. With all other training conditions held constant, all non-empty combinations across the four levels were tested, resulting in a total of 15 placement schemes. The results of these comparisons are summarized in **Table 4**.

**Table 4:** Summary of comparative experiments on multi-level C3k2-UIB.

Model	Level				P	R	mAP@0.50	mAP@0.50-0.95
	P2	P3	P4	P5				
1	✓				0.870	0.859	0.928	0.877
2		✓			0.885	0.892	0.944	0.890
3			✓		0.917	0.804	0.932	0.878
4				✓	0.859	0.902	0.933	0.887
5	✓	✓			0.885	0.894	0.944	0.886
6	✓		✓		0.851	0.898	0.930	0.882
7	✓			✓	0.916	0.852	0.934	0.883
8		✓	✓		0.884	0.859	0.924	0.873
9		✓		✓	0.876	0.835	0.921	0.874
10			✓	✓	0.913	0.847	0.947	0.891
11	✓	✓	✓		0.823	0.896	0.929	0.878
12	✓	✓		✓	0.865	0.864	0.930	0.878
13	✓		✓	✓	0.929	0.835	0.938	0.889
14		✓	✓	✓	0.860	0.884	0.930	0.877
15	✓	✓	✓	✓	0.917	0.896	0.946	0.897

In the single-layer configuration, introducing C3k2-UIB only at the P3 layer yielded the best overall performance, with precision, recall, mAP@0.50, and mAP@0.50-0.95 values of 0.885, 0.892, 0.944, and 0.890, respectively. When C3k2-UIB was used only on Layer P4, precision reached 0.917, but recall dropped to 0.804, indicating that this configuration produced relatively conservative predictions and increased the number of missed targets. In contrast, when used only on Layer P5, recall reached 0.902—the highest value among the single-layer configurations—but precision was only 0.859. These differences indicate that features at different layers play distinct roles in tomato localization and ripeness classification.

In the two-layer configuration, the P4 + P5 combination achieved the highest mAP@0.50 of 0.947, with a precision of 0.947; however, its recall and mAP@0.50-0.95 were 0.847 and 0.891, respectively. In comparison, when C3k2-UIB was simultaneously applied at all four levels (P2, P3, P4, and P5), the mAP@0.50 reached 0.946, which was only 0.1 percentage points lower than that of the P4 + P5 combination. However, recall increased to 0.896, representing an improvement of 4.9 percentage points, while mAP@0.50-0.95 increased to 0.897, the highest value among all layer configurations.

These results indicate that deploying C3k2-UIB only in deeper layers can improve detection performance at lower IoU thresholds, but may reduce object detection rates and bounding box localization stability under high IoU conditions. Simultaneously deploying C3k2-UIB across layers P2 through P5 enables a more balanced feature representation that integrates spatial details from shallow layers, local structures from intermediate layers, and semantic information from deep layers. Therefore, the final YOLO26-CUH adopts a full-level deployment scheme comprising P2, P3, P4, and P5.

### 3.5 Independent cross-domain validation

To evaluate the model’s generalization ability under domain shifts involving tomato variety, fruit size, morphology, and environmental conditions, an independent external validation was conducted using a cherry tomato dataset that was not used in model training, hyperparameter selection, or confidence threshold adjustment. The results on the Provence tomato internal test set and the cherry tomato external validation set are shown in **Table 5**. In the table, dataset ID 1 represents the cherry tomato external independent validation set, which was not used in model training or parameter tuning and was employed to test the model’s cross-domain generalization ability; ID 2 represents the Provence tomato internal test set, used to evaluate the model’s baseline detection performance within the same variety.

**Table 5:** Summary of comparative experiments on different datasets.

Model	Datasets		P	R	mAP@0.50	mAP@0.50-0.95
	1	2				
YOLO26n	✓		0.663	0.696	0.755	0.615
YOLO26-CUH	✓		0.700	0.780	0.808	0.656
YOLO26n		✓	0.850	0.845	0.927	0.878
YOLO26-CUH		✓	0.917	0.896	0.946	0.897

On the Provence tomato internal test set, YOLO26n achieved precision, recall, mAP@0.50, and mAP@0.50-0.95 values of 0.850, 0.845, 0.927, and 0.878, respectively. The corresponding metrics for YOLO26-CUH reached 0.917, 0.896, 0.946, and 0.897, representing improvements of 6.7, 5.1, 1.9, and 1.9 percentage points, respectively.

When both models were directly applied to the independent cherry tomato dataset, their detection performance declined, indicating that variations in fruit size, shape, surface texture, and image composition caused a significant shift in the data distribution. On the external dataset, YOLO26n achieved precision, recall, mAP@0.50, and mAP@0.50-0.95 values of 0.663, 0.696, 0.755, and 0.615, respectively. Under the same conditions, YOLO26-CUH achieved corresponding values of 0.700, 0.780, 0.808, and 0.656, representing improvements of 3.7, 8.4, 5.3, and 4.1 percentage points over the baseline model, respectively.

Notably, the improvement in recall was greater than that in precision, indicating that the primary advantage of YOLO26-CUH under the external domain shift lies in reducing missed detections. From the internal test set to the external validation set, the mAP@0.50 of YOLO26n decreased by 17.2 percentage points, whereas that of YOLO26-CUH decreased by 13.8 percentage points, representing a 3.4-percentage-point reduction in performance degradation. For mAP@0.50-0.95, the internal-to-external performance gaps of YOLO26n and YOLO26-CUH were 26.3 and 24.1 percentage points, respectively.

The results indicate that YOLO26-CUH can mitigate, to some extent, the performance degradation caused by differences in data distribution across varieties, but it cannot yet completely eliminate the domain gap between large-fruited Provence tomatoes and small-fruited tomatoes. The remaining performance gap on the external dataset also suggests that cross-domain ripeness detection remains more challenging than internal testing within the same data distribution.

### 3.6 Robustness to global brightness perturbations

To verify the model’s ability to adapt to changes in lighting conditions, the original test images were processed using brightness coefficients ranging from 0.5 to 1.5, with a step size of 0.1. A brightness coefficient less than 1 indicates that the image was darkened, while a coefficient greater than 1 indicates that the image was brightened. The fully trained YOLO26n and YOLO26-CUH models were evaluated directly on test sets with varying brightness levels, without any retraining, fine-tuning, or parameter adjustments.

As shown in **Figure 7**, both models achieved high detection performance near the original luminance coefficient of 1.0. When image brightness decreased or increased substantially, both mAP@0.50 and mAP@0.50-0.95 declined to varying degrees, indicating that drastic illumination changes affected tomato skin color information, object edges, and bounding-box localization. Particularly under extreme brightness conditions, the loss of detail in dark areas or saturation in highlight regions further reduces the discriminative differences between adjacent maturity stages.

**Figure 7:**
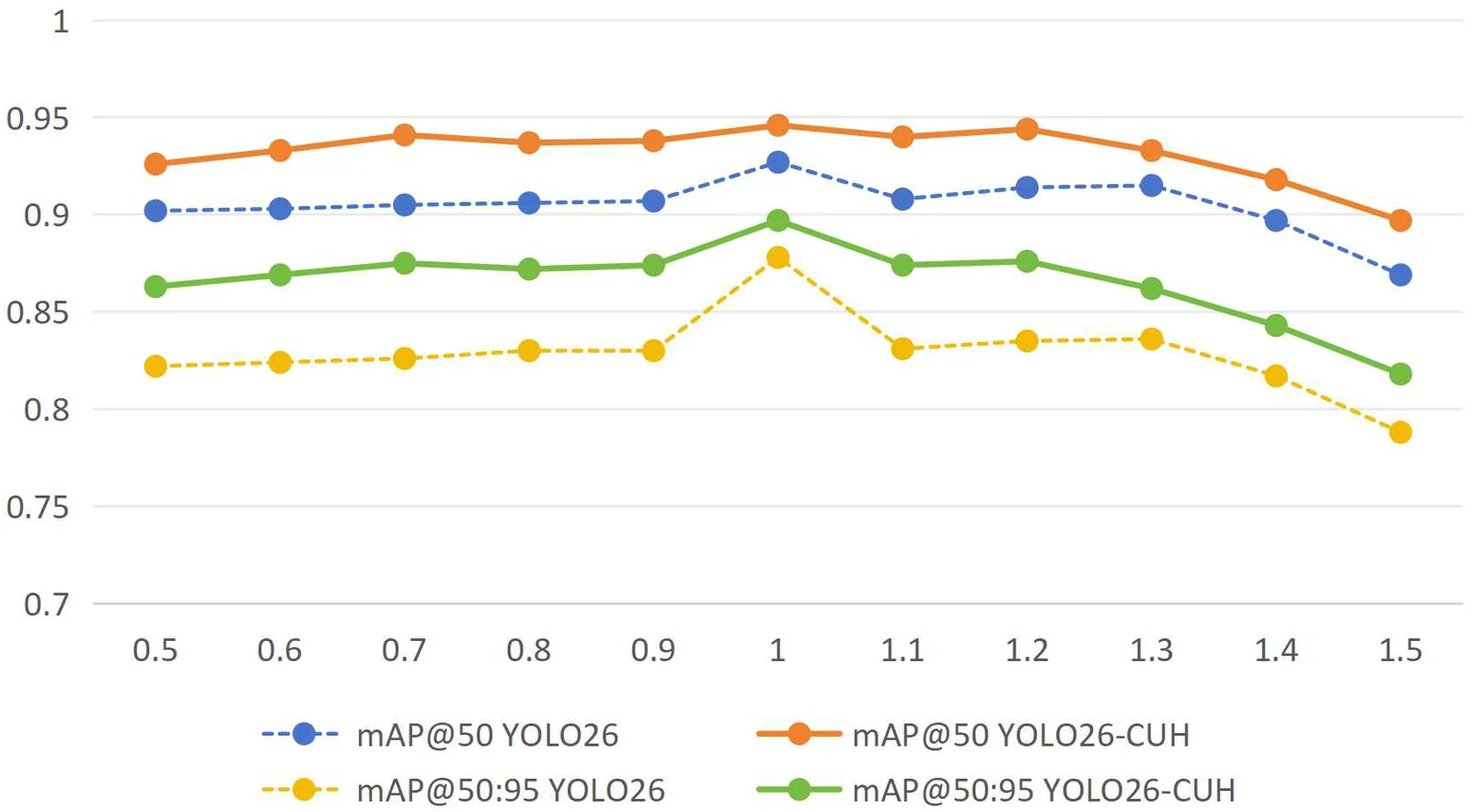
Detection performance of YOLO26n and YOLO26-CUH under different lighting conditions.

Across the entire range of brightness coefficients from 0.5 to 1.5, YOLO26-CUH consistently outperformed the original YOLO26n in terms of both mAP@0.50 and mAP@0.50-0.95. This advantage was not limited to standard lighting conditions but persisted under both underexposed and overexposed conditions, indicating that the performance improvement of the proposed model is not confined to a single exposure level. These results indicate that the complete YOLO26-CUH architecture provides greater stability than the YOLO26n baseline under controlled global brightness perturbations.

Nevertheless, both models still exhibit performance degradation under the most extreme underexposure and overexposure conditions. Therefore, while YOLO26-CUH improves the model’s resistance to brightness variations, it has not yet completely eliminated the effects caused by severe underexposure and overexposure. It should be noted that this experiment simulates only controlled global brightness changes and does not fully represent complex greenhouse illumination conditions, such as strong backlighting, localized shadows, specular reflections, or nighttime supplemental lighting. Therefore, the results should be interpreted as evidence of improved robustness to global brightness perturbations rather than comprehensive illumination robustness.

## 4 Discussion

### 4.1 Analysis of classification errors by model type

The training process and normalized confusion matrix for YOLO26-CUH are shown in **Figure 8**. In the early stages of training, the bounding box regression loss, classification loss, and distribution focus loss all decreased rapidly, while precision, recall, and mAP metrics increased simultaneously, indicating that the model was able to quickly learn the location and ripeness features of tomato targets. As training progressed, the training and validation set losses gradually stabilized, and after approximately 150–200 epochs, all evaluation metrics entered a relatively stable phase. Although there were minor fluctuations in the later stages of training, there was no sustained increase in validation loss or significant decline in detection accuracy, indicating that the model exhibited good convergence and showed no obvious signs of overfitting. The final model achieved precision, recall, mAP@0.50, and mAP@0.50-0.95 values of 0.917, 0.896, 0.946, and 0.897, respectively.

**Figure 8:**
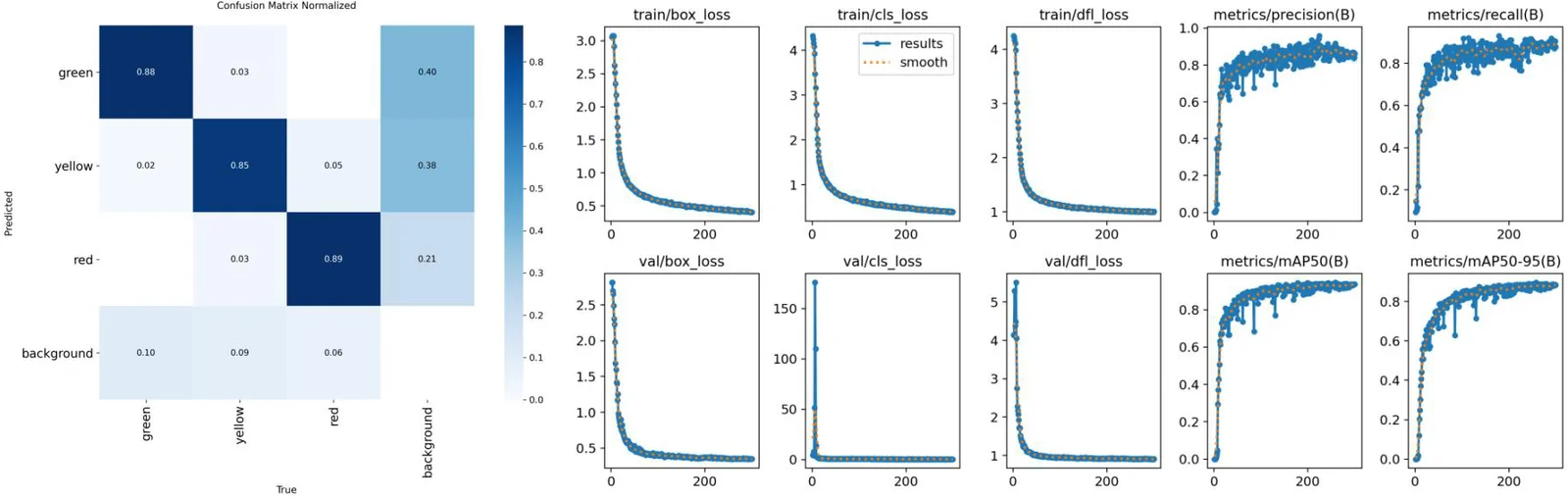
Convergence characteristics and classification error confusion matrix of the YOLO26-CUH model.

Based on the normalized confusion matrix, the correct classification rates for unripe, color-changing, and ripe tomatoes were 0.88, 0.85, and 0.89, respectively, indicating that the model demonstrates relatively stable classification performance across all three ripeness stages. Among them, ripe tomatoes had the highest correct classification rate, suggesting that there is a distinct color difference between the red fruit skin and the background, allowing the model to easily extract discriminative features. The correct identification rate for unripe tomatoes was 0.88; however, 0.10 of the actual unripe targets were still classified as background. This is primarily due to the similarities in color, texture, and local edges between green fruits and leaves.

The correct identification rate for color-changing tomatoes was relatively low at 0.85. Among the true color-changing tomatoes, 0.03 were predicted to be unripe, and another 0.03 were predicted to be ripe, indicating that this category is primarily confused with adjacent maturity stages. This is because the color-changing stage represents a transitional state between green and red, where the surface of a single fruit may simultaneously contain green, yellow, and light red areas, resulting in a lack of completely clear visual boundaries between categories. Furthermore, 0.09 of the true color-changing targets were predicted as background, indicating that missed detections may still occur when objects are occluded, are small in scale, or have inconspicuous localized colored areas.

Among the true ripe tomatoes, 0.05 were predicted as color-changing, and only 0.06 were classified as background; the missed detection rate for this category is lower than that for the unripe and color-changing categories. This suggests that YOLO26-CUH has good detection capability for ripe fruits, but may still confuse them with the color-changing stage when coloring is incomplete or affected by lighting conditions. The results in the “Background” column show that background-related false positives are primarily assigned to the “unripe” and “color-changing” categories, accounting for 0.40 and 0.38, respectively, while the “ripe” category accounts for 0.21. It should be noted that these values represent the distribution of background-related false positives across different prediction classes, not the model’s absolute false positive rate for all background regions. The fact that the “unripe” and “color-changing” classes generate more background false positives further illustrates that green leaves, branches, and localized changes in lighting are prone to producing feature responses similar to those of unripe or color-changing fruits.Quantitatively, the confusion matrix indicates that background-related errors occur more frequently for unripe and color-changing tomatoes than for ripe tomatoes, which is consistent with the greater visual similarity between green fruits and foliage and the reduced visible information under occlusion. However, the present study does not separately quantify false positives and missed detections according to occlusion level, target scale, or fruit density, and such condition-specific error analysis will be considered in future work.

Overall, the training curves indicate that YOLO26-CUH converges stably, while the confusion matrix shows that model errors are primarily concentrated in classification confusion between color-changing tomatoes and adjacent maturity stages, as well as in missed detections and false positives between unripe fruits and green backgrounds. This aligns with the continuous color changes during tomato ripening and the complex characteristics of the greenhouse background, and it also indicates that further improving the model’s ability to distinguish transitional ripening stages and suppress green background noise remains a key focus for future optimization.

### 4.2 Visual comparison of detection performance between the baseline and improved models

To further compare the actual detection performance of different models in complex greenhouse environments, four sets of representative test images were selected for visual analysis. The selected scenarios included situations such as occlusion by branches and leaves, dense overlapping of fruits, variations in target size, local reflections, and the coexistence of fruits at different stages of ripeness. All models used the same input images and inference settings to ensure the comparability of the results. In **Figure 9**, detection boxes of different colors represent unripe, color-changing, and ripe tomatoes, respectively; the values above the boxes indicate the predicted confidence levels for the corresponding categories.

**Figure 9:**
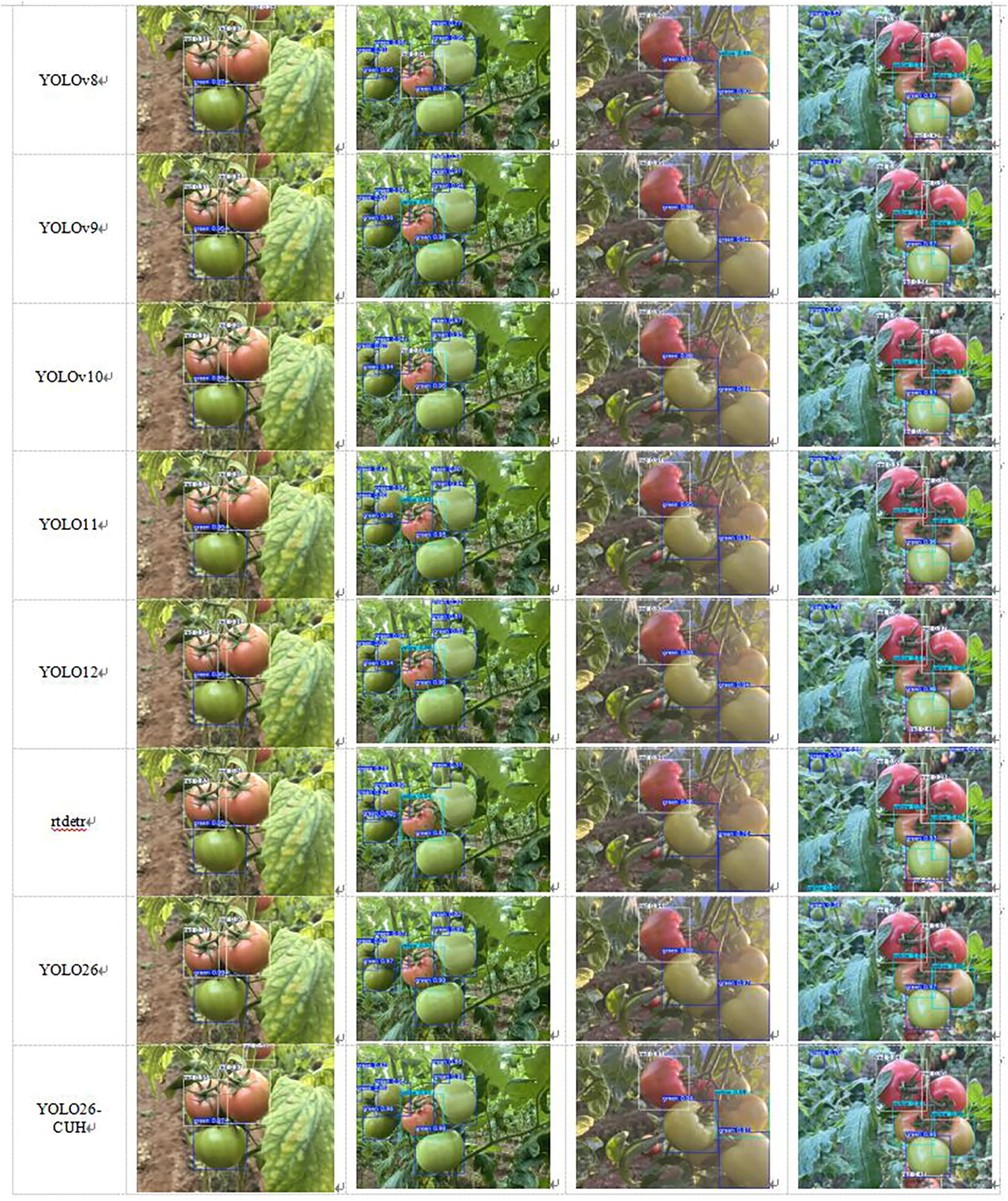
Visual comparison of tomato ripeness detection performance across different detection models in complex greenhouse scenarios.

Looking at the overall results, YOLOv8n, YOLOv9t, YOLOv10n, YOLO11n, YOLO12n, RT-DETR, and YOLO26n were all able to detect large-scale fruits with complete outlines and low occlusion levels, indicating that these models possess basic tomato ripeness detection capabilities. However, in scenarios with dense fruit distribution or severe occlusion by branches and leaves, the differences among the models become more pronounced. Some models fail to detect small targets where only a partial area is exposed, or exhibit issues such as incomplete bounding box coverage, overlapping boundaries between adjacent fruits, and reduced prediction confidence. When unripe tomatoes are similar in color to the leaves, the green texture in the background further blurs the object edges, making it difficult for some models to reliably distinguish fruits from the surrounding branches and leaves.

In images containing fruits at multiple stages of ripeness, the color of color-changing tomatoes falls between green and red, and their visual features exhibit strong continuity, making them more prone to confusion with adjacent ripeness levels. Although some of the compared models can accurately locate the fruits, their performance in category classification and confidence estimation is inconsistent. In contrast, YOLO26-CUH maintains relatively complete object coverage for unripe, color-changing, and ripe tomatoes, with detection boxes showing a higher degree of alignment with the boundaries of the fruit bodies. In scenes with densely packed objects, the improved model can effectively distinguish between adjacent fruits and provides accurate predictions for partially occluded and small-scale objects.

The visualization results of YOLO26-CUH are generally consistent with the quantitative experimental findings. In the comparative experiments, this model achieved the highest recall, mAP@0.50, and mAP@0.50-0.95, indicating that its performance advantages primarily lie in reducing false negatives in complex backgrounds while maintaining stable ripeness classification and object localization. Although low-confidence predictions may still occur in cases of extreme occlusion or when the visible region of a target is too small, the overall results indicate that YOLO26-CUH demonstrates better adaptability to interference from branches and leaves, fruit overlap, and scale variations.

### 4.3 Comparative analysis of heat maps for model feature attention regions

To further analyze the feature responses of different models to the target regions in tomato images, we used the prediction-region-guided Grad-CAM++ method to perform a visual comparison of four sets of typical greenhouse scenes. The results are shown in **Figure 10**. The test images include scenarios such as occlusion by branches and leaves, dense fruit distribution, the coexistence of fruits at different stages of ripeness, and uneven illumination. In the figure, red and yellow regions indicate feature responses that contribute significantly to the model’s predictions, while blue regions indicate lower contributions. All models used the same input images, normalization methods, color mapping, and post-processing parameters to ensure the comparability of the visualization results.

**Figure 10:**
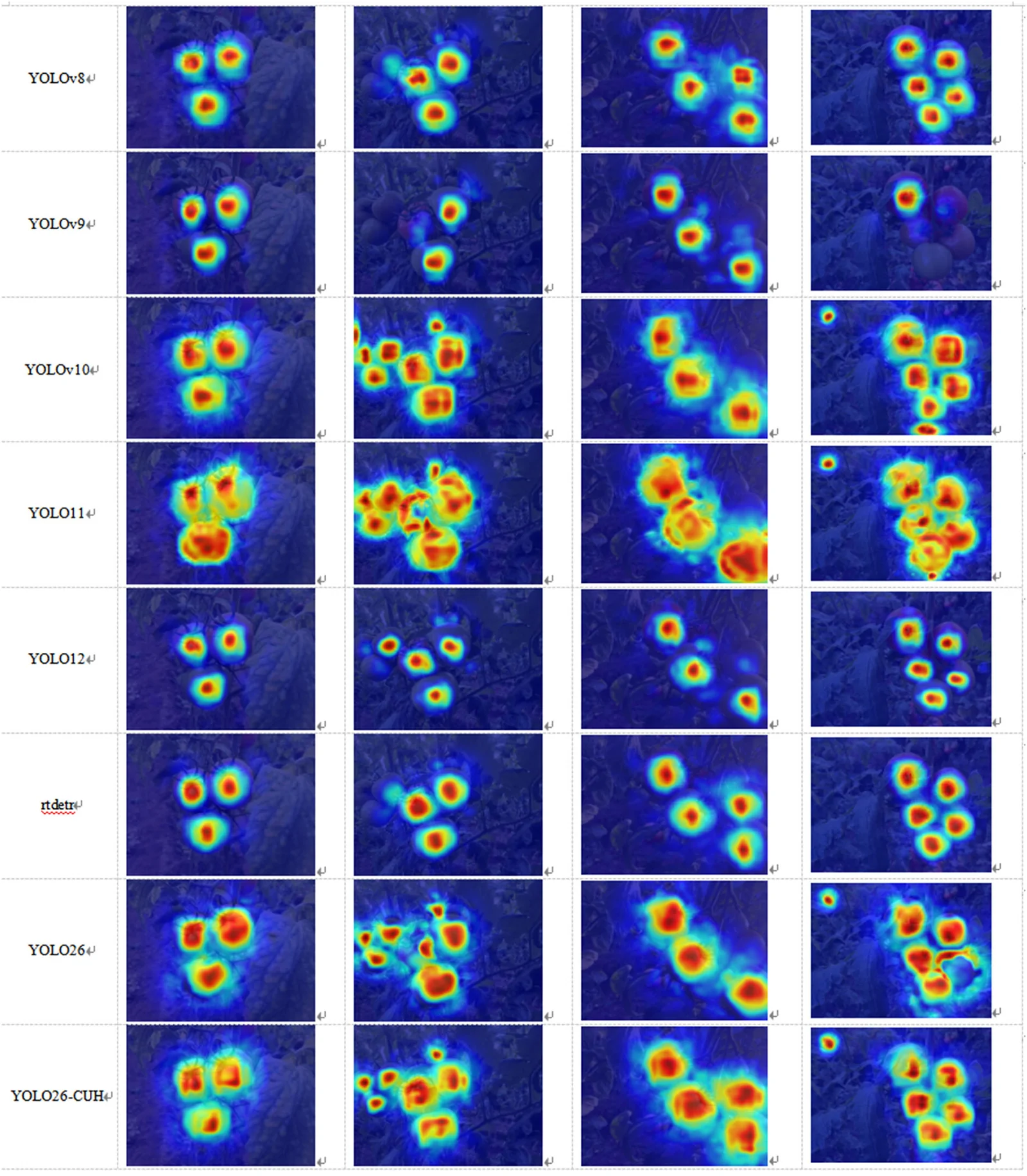
Comparison of attention heatmaps for tomato target region features across different detection models.

Overall, all models are able to localize high-response regions near tomato fruits to some extent, but there are significant differences in response completeness, target coverage, and background suppression capabilities. YOLOv8n, YOLO11n, and YOLO12n can generate relatively concentrated high-response regions in scenes with few targets and well-defined contours; however, in scenes with dense clusters of fruits or occlusions, the responses for some small-scale and low-contrast targets are weak. YOLOv9t’s response regions are relatively sparse, and some occluded fruits do not form distinct hotspots, indicating that its feature responses tend to concentrate on targets with more prominent colors or contours. YOLOv10n and RT-DETR can cover most major fruits, but some responses spread toward the fruit stems, leaves, and surrounding areas, and the distinction between targets and the background remains insufficiently clear.

The original YOLO26n exhibits strong responses to tomato bodies but displays noticeable response diffusion and local discontinuities in dense scenes. Some red and yellow regions extend into the gaps between fruits, branches, leaves, and the surrounding background, suggesting that shallow color and texture information may be blended with background features. When multiple fruits occlude one another or are at similar stages of ripeness, this diffuse response may weaken the model’s ability to distinguish individual instance boundaries and local color variations. In contrast, YOLO26-CUH is able to form relatively complete and continuous high-response regions for the main tomatoes in all four sets of images. In scenes with densely packed fruits, the model can simultaneously focus on multiple adjacent targets, rather than generating strong responses only to fruits with larger areas or more distinctive colors; for fruits partially obscured by branches and leaves or located in shadows, it still retains relatively distinct feature responses. At the same time, YOLO26-CUH’s high-response regions are primarily distributed around the main body of the fruit, localized color regions, and near the edges, while responses to leaves and other irrelevant background areas are relatively weak, indicating that the improved model possesses better target focusing and background suppression capabilities.

The observed response patterns are consistent with the overall design objective of YOLO26-CUH. Compared with the baseline model, YOLO26-CUH produces more concentrated responses within tomato regions and relatively weaker responses in surrounding background areas, indicating a greater tendency to focus on fruit-related features under complex greenhouse conditions. These visualization results are consistent with the performance trends observed in the ablation experiments.

### 4.4 Limitations and future research directions

This paper addresses the task of tomato ripeness detection in complex greenhouse environments. It introduces improvements to YOLO26n in terms of feature enhancement, lightweight feature extraction, and cross-layer fusion architecture. The effectiveness of YOLO26-CUH is validated through ablation experiments, multi-model comparisons, illumination perturbation tests, independent cross-domain validation, and feature response visualization. However, the self-built dataset was primarily collected from the same plastic-film greenhouse, during the same growing season, and using a single Provence tomato variety. Although it covers typical scenarios such as foliage occlusion, dense fruit distribution, and uneven illumination, it still lacks sufficient coverage for strong backlighting, severe overexposure and underexposure, greenhouse film reflections, nighttime supplemental lighting, and variations across different seasons and cultivation sites. In illumination perturbation experiments, both models exhibited performance degradation under extreme brightness conditions, indicating that YOLO26-CUH remains somewhat sensitive to severe changes in illumination; its robustness in more complex production environments requires further validation.

This paper classifies tomatoes into three stages: unripe, color-changing, and ripe; however, tomato skin coloration is essentially a continuous process, and the color-changing stage, in particular, lacks completely distinct visual boundaries between adjacent categories. In the experiments, the precision for the color-changing category was lower than for other categories, and the confusion matrix also showed that it was primarily confused with the unripe and ripe categories. The current model relies solely on single-frame RGB images for detection and has not yet incorporated temporal information—such as color changes, growth rates, and morphological changes—during the continuous growth process of the fruit; consequently, it is difficult to use growth patterns to assist in judging borderline samples. Although YOLO26-CUH outperformed the baseline models on the independent cherry tomato dataset, its mAP@0.50 and mAP@0.50-0.95 decreased from 0.946 and 0.897 on the internal test set to 0.808 and 0.656, respectively, indicating that substantial domain shifts remained across tomato varieties, fruit shapes and sizes, and image distributions. For extreme samples—such as those with severe leaf occlusion, multiple tightly overlapping fruits, or only small localized areas exposed—the model may still experience missed detections, overlapping bounding boxes, or misclassification of ripeness. This paper uses object detection bounding boxes to describe fruit positions and has not yet achieved pixel-level separation of densely overlapping fruits. Furthermore, the inference speed of 93.9 FPS was obtained on an RTX 4090 GPU; the model’s accuracy, latency, power consumption, and long-term stability have not yet been systematically verified on low-power edge devices or in real-world inspection or harvesting platforms.

Future research will further expand the dataset to include multiple seasons, multiple growing sites, and multiple tomato varieties, incorporating samples under strong backlighting, reflections, extreme shading, nighttime lighting, and various camera conditions. By combining domain generalization or unsupervised domain adaptation methods, the model’s adaptability to changes in real production environments will be enhanced. Additionally, continuous video or multi-temporal images can be incorporated to establish temporal correlations between fruit color, morphology, and growth processes, thereby aiding in the classification of samples during color-change stages and at the boundaries of maturity. To address issues of dense overlap and severe occlusion, an instance segmentation or fruit contour refinement branch can be added to achieve pixel-level separation of fruit regions. Combined with uncertainty estimation, this approach can output confidence scores or continuous ripeness scores for hard-to-distinguish transitional stages. In terms of engineering applications, edge deployment tests should be conducted on actual greenhouse inspection robots or harvesting platforms. Model pruning, quantization, and operator optimization should be employed to reduce inference latency and power consumption, thereby comprehensively evaluating the detection performance and practical value of YOLO26-CUH in real production environments.In addition, although representative examples of occlusion, small targets, and dense fruit distributions were included in the current evaluation, systematic quantitative analyses stratified by occlusion level, target scale, and target density were not conducted and will be considered in future work.

## 5 Summary

This paper addresses challenges in tomato ripeness detection under complex greenhouse environments, such as the similarity in color between unripe fruits and leaves, subtle differences during the color-change stage, obstruction by branches and leaves, dense overlapping of fruits, and the tendency for small target features to fade. To address these issues, the authors constructed a dataset comprising 5, 296 images of Provence tomatoes across three ripeness stages and proposed the YOLO26-CUH tomato ripeness detection model, using YOLO26n as the baseline.

Quantitative experimental results show that YOLO26-CUH has 2, 380, 194 parameters, requires 6.2 GFLOPs, and achieves an inference speed of 93.9 FPS on a desktop platform. Its precision, recall, mAP@0.50, and mAP@0.50-0.95 reach 0.917, 0.896, 0.946, and 0.897, respectively. Compared to the original YOLO26n, these four metrics improved by 6.7, 5.1, 1.9, and 1.9 percentage points, respectively, while the number of parameters increased by only 4, 773, indicating that the model’s performance improvement does not rely on a significant increase in network size.

Ablation experiments revealed distinct functional contributions and non-additive interactions among C2PSA-CC, C3k2-UIB, and HV-LCA. Specifically, C2PSA-CC enhanced the expression of ripeness-related features without increasing computational complexity; C3k2-UIB reduced the number of parameters to 2, 209, 845 and computational load to 4.9 GFLOPs while maintaining detection accuracy close to the baseline; HV-LCA primarily improved recall performance, indicating its contribution to reducing missed detections of low-contrast and occluded targets. Layer-wise experiments on C3k2-UIB further demonstrated that simultaneously replacing the structures at the P2, P3, P4, and P5 layers yielded the highest mAP@0.50-0.95 and a better balance between precision and recall. Therefore, the final model adopted this full-hierarchy configuration. In independent cross-domain validation using the cherry tomato dataset, YOLO26-CUH achieved precision, recall, mAP@0.50, and mAP@0.50-0.95 values of 0.700, 0.780, 0.808, and 0.656, respectively. These results represented improvements of 3.7, 8.4, 5.3, and 4.1 percentage points over the baseline model, respectively, indicating that the proposed model could mitigate performance degradation caused by differences in fruit shape, scale, and varietal characteristics and was particularly effective in reducing missed detections under the external domain shift. However, the external validation results remained lower than those obtained on the internal Provence tomato test set, suggesting that differences in data distributions across varieties and acquisition environments had not yet been fully addressed. Illumination perturbation experiments showed that, across the brightness coefficient range of 0.5–1.5, YOLO26-CUH consistently outperformed the original YOLO26n in terms of both mAP@0.50 and mAP@0.50-0.95, demonstrating greater stability under controlled global brightness perturbations. Nevertheless, its performance still declined under severe underexposure and overexposure. Grad-CAM++ visualizations were consistent with the quantitative performance trends and showed more concentrated responses within tomato regions and relatively weaker responses in surrounding background areas.

In summary, while maintaining a largely unchanged parameter scale, YOLO26-CUH improves the accuracy, recall, robustness to global brightness perturbations, and cross-domain generalization capability for tomato ripeness detection, while sustaining a desktop inference speed of 93.9 FPS. It provides a visual perception method for intelligent monitoring, ripeness grading, and selective harvesting of greenhouse tomatoes. Future research will further expand the dataset to include multiple seasons, multiple sites, and multiple varieties; incorporate samples under extreme lighting conditions and with severe occlusion; introduce growth-timing information, instance segmentation, and uncertainty estimation; and conduct comprehensive deployment validation on low-power edge devices and real-world inspection and harvesting platforms to further enhance the model’s reliability and engineering applicability in real production environments.

## Data Availability

The raw data supporting the conclusions of this article will be made available by the authors, without undue reservation.
